# Development of a monoclonal antibody and a lateral-flow device for the rapid detection of a Mucorales-specific biomarker

**DOI:** 10.3389/fcimb.2023.1305662

**Published:** 2023-12-08

**Authors:** Christopher R. Thornton, Genna E. Davies, Laura Dougherty

**Affiliations:** ^1^ Biosciences, Faculty of Health and Life Sciences, University of Exeter, Exeter, United Kingdom; ^2^ ISCA Diagnostics Ltd., Hatherly Laboratories, Exeter, United Kingdom

**Keywords:** Mucorales, mucoromycosis, monoclonal antibody, biomarker, lateral-flow test

## Abstract

Mucoromycosis is a highly aggressive angio-invasive disease of humans caused by fungi in the zygomycete order, Mucorales. While *Rhizopus arrhizus* is the principal agent of mucoromycosis, other Mucorales fungi including *Apophysomyces*, *Cunninghamella*, *Lichtheimia*, *Mucor*, *Rhizomucor* and *Syncephalastrum* are able to cause life-threatening rhino-orbital-cerebral, pulmonary, gastro-intestinal and necrotising cutaneous infections in humans. Diagnosis of the disease currently relies on non-specific CT, lengthy and insensitive culture from invasive biopsy, and time-consuming histopathology of tissue samples. At present, there are no rapid antigen tests that detect Mucorales-specific biomarkers of infection, and which allow point-of-care diagnosis of mucoromycosis. Here, we report the development of an IgG2b monoclonal antibody (mAb), TG11, which binds to extracellular polysaccharide (EPS) antigens of between 20 kDa and 250 kDa secreted during hyphal growth of Mucorales fungi. The mAb is Mucorales-specific and does not cross-react with other yeasts and molds of clinical importance including *Aspergillus*, *Candida*, *Cryptococcus*, *Fusarium*, *Lomentospora* and *Scedosporium* species. Using the mAb, we have developed a Competitive lateral-flow device that allows rapid (30 min) detection of the EPS biomarker in human serum and bronchoalveolar lavage (BAL), with a limit of detection (LOD) in human serum of ~100 ng/mL serum (~224.7 pmol/L serum). The LFD therefore provides a potential novel opportunity for detection of mucoromycosis caused by different Mucorales species.

## Introduction

Mucoromycosis (Borman and Johnson, 2023) is a highly destructive angio-invasive disease of humans caused by zygomycete fungi in the order Mucorales (Thornton, 2020), recently characterised as a high priority group in the World Health Organisation’s fungal priority pathogens list (WHO, 2022). The disease encompasses debilitating rhino-orbital-cerebral mucoromycosis (ROCM), and pulmonary, cutaneous, gastro-intestinal and disseminated infections (Petrikkos et al., 2012; Ganesan et al., 2019; Jeong et al., 2019; Thornton, 2023) which, prior to the COVID-19 pandemic, were typically seen in patients with haematological malignancies (Miller et al., 2020), in bone marrow and solid organ transplant recipients (Roden et al., 2005; Song et al., 2017; Miller et al., 2020; Skiada et al., 2020) and in individuals with poorly controlled diabetes mellitus (DM), a major independent risk factor for the disease (Corzo-León et al., 2018; Skiada et al., 2020; Thornton, 2023). However, during the second wave of the pandemic in India, there was a dramatic increase in ROCM in patients with severe SARS-CoV-2 infection, exacerbated by a high background prevalence of DM and the overuse of anti-inflammatory corticosteroids (John et al., 2021; Rodriguez-Morales et al., 2021; Sen et al., 2021). While *Rhizopus arrhizus* is the principal cause of mucoromycosis worldwide (Prakash and Chakrabarti, 2019; Davies and Thornton, 2022), and was responsible for a large number of cases of COVID-19-associated mucoromycosis (CAM) in India and other countries worldwide (Prakash and Chakrabarti, 2019; Prakash and Chakrabarti, 2021; Hoenigl et al., 2022), Mucorales fungi other than *R. arrhizus* are able to cause mucoromycosis, including species in the genera *Apophysomyces*, *Cunninghamella*, *Lichtheimia*, *Mucor*, *Rhizomucor*, *Saksenaea*, and *Syncephalastrum* (Álvarez et al., 2009; Gomes et al., 2011; Zaki et al., 2014; Jeong et al., 2019; Walther et al., 2019; Skiada et al., 2020; Thornton, 2023).

Mucoromycosis is associated with high rates of mortality, with an overall all-cause mortality rate of 54% (Roden et al., 2005). Furthermore, survivors of ROCM are often left with severe facial disfigurement due to the aggressive surgery needed to contain rapidly progressive infections (Sen et al., 2021). The disease is especially problematic in low- to middle-income countries (LMIC), where limited access to well-resourced and appropriately-equipped diagnostic facilities delays diagnosis and treatment (Rudramurthy et al., 2021; Thornton, 2023). There is therefore an urgent need for simple, rapid and accurate diagnostic tests for the disease that can be performed at point-of-care. Lateral-flow immunoassays are ideally suited to point-of-care detection of fungal infections in resource-limited settings (Thornton, 2020; Osaigbovo and Bongomin, 2021; Thornton, 2023), and might help to improve the speed and accuracy of mucoromycosis detection compared to insensitive and time-consuming culture and histopathology, the cornerstones of detection in LMIC countries (Rudramurthy et al., 2021; Thornton, 2023).

At present, there are no antigen biomarker tests which allow rapid, sensitive and specific detection of Mucorales species (Skiada et al., 2020; Lamoth, 2023), and their differentiation from other fungal pathogens such as *Aspergillus*, *Candida*, *Cryptococcus*, *Fusarium* and *Scedosporium* (Marques et al., 2011; Obradovic-Tomasev et al., 2014; Skiada et al., 2020; Marino et al., 2023). Despite this, we recently reported the development of a monoclonal antibody (mAb), KC9, specific to *R. arrhizus*, and its incorporation into a lateral-flow device (KC9-LFD) for rapid detection of an extracellular polysaccharide (EPS) biomarker of the pathogen in human serum and bronchoalveolar lavage (BAL) fluid (Davies and Thornton, 2022). While sensitive and simple to perform, the test detects *R. arrhizus* only, and so is unable to detect the other Mucorales fungi capable of causing mucoromycosis in humans.

In this paper, we report the development of a murine mAb, TG11, and a Competitive LFD for the detection of a Mucorales-specific EPS biomarker in human serum and BAL. We show that the pan-Mucorales test, when combined with a Cube reader, has a limit of detection (LOD) of ~100 ng/mL serum (~224.7 pmol/L serum). This is the first time, to the best of our knowledge, that a Mucorales-specific mAb has been developed and used in a rapid point-of-care test (POCT) for detection of these life-threatening pathogens.

## Materials and methods

### Ethics statement

Hybridoma work described in this study was conducted under a UK Home Office Project License, and was reviewed by the institution’s Animal Welfare Ethical Review Board (AWERB) for approval. The work was carried out in accordance with The Animals (Scientific Procedures) Act 1986 Directive 2010/63/EU, and followed all the Codes of Practice which reinforce this law, including all elements of housing, care, and euthanasia of the animals.

### Fungal cultures

Fungi (**Table 1**) were cultured on malt extract agar (MEA; 70145, Sigma) or oatmeal agar (OA; O3506, Sigma). Media were autoclaved 121°C for 15 min prior to use, and fungi were grown at 30°C or 37°C. To induce sporulation in *Apophysomyces* spp., the fungi were grown on autoclaved Czapek Dox agar (CDA; 70185, Sigma) at 37°C.

**Table 1:** Details of the fungi used in this study and results of TG11-LFD and KC9-LFD tests of culture filtrates.

Species	Isolate Number	Source^1^	TG11 a.u.^2^	KC9 a.u.^2^	
*Absidia glauca*	2	CRT	51.5	776.3	
*Absidia spinosa*	3	CRT	69.6	781.1	
*Actinomucor elegans* var. *kuwaitensis*	117697	CBS	13.5	770.2	
*Apophysomyces elegans*	477.78	CBS	240.9	789.5	
*Apophysomyces mexicanus*	136361	CBS	244.7	780.1	
*Apophysomyces ossiformis*	125533	CBS	109.0	775.3	
*Apophysomyces variabilis*	658.93	CBS	250.6	801.4	
*Aspergillus fumigatus*	Af293	FGSC	427.3	746.0	
*Aspergillus flavus*	144B	CRT	418.0	791.1	
*Aspergillus nidulans*	A4	FGSC	430.1	741.2	
*Aspergillus niger*	102.4	CBS	403.8	774.7	
*Aspergillus terreus* var. *terreus*	601.65	CBS	402.2	782.0	
*Candida albicans*	SC5314	SB	435.4	774.2	
*Cryptococcus neoformans*	8710	CBS	450.0	774.4	
*Cunninghamella bertholletiae*	151.80	CBS	3.9	768.0	
*Fusarium oxysporum*	167.3	CBS	400.1	770.7	
*Fusarium solani*	224.34	CBS	434.1	779.3	
*Lichtheimia corymbifera*	109940	CBS	14.2	797.6	
*Lichtheimia corymbifera*	120580	CBS	19.8	786.0	
*Lichtheimia hyalospora*	146576	CBS	50.0	765.7	
*Lichtheimia ornata*	142195	CBS	10.2	787.7	
*Lichtheimia ramosa*	112528	CBS	13.5	784.4	
*Lichtheimia ramosa*	124197	CBS	38.8	776.4	
*Lichtheimia ramosa*	2845	NCPF	7.7	789.0	
*Lomentospora prolificans*	3.1	CRT	433.3	766.6	
*Mucor ardhlaengiktus*	126271	CBS	7.3	743.8	
*Mucor circinelloides* f. *circinelloides*	123973	CBS	6.6	777.8	
*Mucor circinelloides*	124429	CBS	0.6	790.5	
*Mucor circinelloides*	B5-2	CRT	15.0	786.5	
*Mucor circinelloides* f. *circinelloides*	120582	CBS	0.0	781.0	
*Mucor circinelloides* f. *circinelloides*	E2A	CRT	0.8	774.0	
*Mucor indicus*	120.08	CBS	8.8	761.9	
*Mucor irregularis*	103.93	CBS	1.9	750.1	
*Mucor plumbeus*	96	CRT	17.7	762.6	
*Mucor racemosus* f. *racemosus*	112382	CBS	16.6	768.3	
*Mucor velutinosus*	126272	CBS	3.9	769.4	
*Phycomyces nitens*	133	CBS	0.8	778.5	
*Rhizomucor pusillus*	120586	CBS	9.6	774.5	
*Rhizomucor pusillus*	120587	CBS	3.7	750.3	
*Rhizopus arrhizus*	T14A	CRT	115.9	25.0	
*Rhizopus arrhizus*	TV4	CRT	85.8	13.0	
*Rhizopus arrhizus*	2634	NCPF	51.7	50.8	
*Rhizopus arrhizus*	111233	CBS	60.1	9.6	
*Rhizopus arrhizus* var. *arrhizus*	112.07	CBS	34.5	24.2	
*Rhizopus arrhizus* var. *arrhizus*	118614	CBS	51.2	10.0	
*Rhizopus arrhizus* var. *delemar*	2601	NCPF	17.3	11.9	
*Rhizopus arrhizus* var. *delemar*	544.80	CBS	30.7	9.3	
*Rhizopus arrhizus* var. *delemar*	607.68	CBS	33.1	19.9	
*Rhizopus azygosporus*	357.93	CBS	15.7	794.8	
*Rhizopus homothallicus*	336.62	CBS	181.1	782.5	
*Rhizopus microsporus* var. *rhizopodiformis*	102277	CBS	6.6	802.1	
*Rhizopus microsporus* var. *rhizopodiformis*	220.92	CBS	41.3	809.9	
*Rhizopus microsporus* var. *rhizopodiformis*	118987	CBS	33.2	848.2	
*Rhizopus oligosporus*	Tempeh	CRT	11.2	783.7	
*Rhizopus schipperae*	138.95	CBS	5.6	790.1	
*Rhizopus stolonifer* var. *stolonifer*	389.95	CBS	7.0	743.9	
*Saksenaea erythrospora*	138279	CBS	57.0	804.7	
*Scedosporium apiospermum*	117467	CBS	401.7	781.0	
*Scedosporium aurantiacum*	121926	CBS	428.5	781.3	
*Scedosporium boydii*	835.96	CBS	476.7	778.4	
*Syncephalastrum racemosum*	155	CRT	3.4	778.4	

^1^CBS; Westerdijk Fungal Biodiversity Institute, The Netherlands. CRT, C. R. Thornton, University of Exeter, UK. NCPF, National Centre for Pathogenic Fungi, Public Health England, UK.

^2^For LFD tests, test (T) line Cube readings in artificial units (a.u.) are the means of two replicate values. The threshold T line value for TG11-LFD test positivity is ≤400 a.u., while the threshold T line value for KC9-LFD test positivity is ≤700 a.u. All TG11-LFD and KC9-LFD tests had control (C) line values of ≥600 a.u.

### Production of hybridomas and screening by indirect ELISA

Extracellular polysaccharides (EPS) were prepared from 6-d-old culture fluids using the method described previously (Davies and Thornton, 2022). For hybridoma production, the immunogen comprised a 1 mg/mL solution of EPS from *Lichtheimia corymbifera* (strain CBS109940), with 6-week-old BALB/c white mice each given four intra-peritoneal injections (300 µL per injection) of immunogen at 2-week intervals, and a single booster injection 5 days before fusion. Hybridoma cells were produced by the method described elsewhere (Thornton, 2001), and monoclonal antibody (mAb)-producing clones identified in indirect ELISA tests by using 20 μg EPS/mL phosphate-buffered saline (PBS; 137 mM NaCl, 2.7 mM KCl, 8 mM Na_2_HPO_4_, 1.5 mM KH_2_PO_4_, pH 7.2) immobilised to the wells of Maxisorp microtiter plates (Nunc) at 50 µL/well. Wells containing immobilised antigen were incubated with 50 µL of mAb hybridoma tissue culture supernatant (TCS) for 1 h, after which wells were washed three times, for 5 min each, with PBST (PBS containing 0.05% (v/v) Tween-20). Goat anti-mouse polyvalent immunoglobulin (G, A, M) peroxidase conjugate (PA1-84388, Invitrogen), diluted 1:5000 in PBST, was added to the wells and incubated for a further hour. The plates were washed with PBST as described, given a final 5 min wash with PBS, and bound antibody visualised by incubating wells with tetramethyl benzidine (TMB) substrate solution for 30 min, after which reactions were stopped by the addition of 3 M H_2_SO_4_. Absorbance values were determined at 450 nm using a microplate reader (infinite F50, Tecan Austria GmbH). Control wells were incubated with tissue culture medium (TCM) containing 10% (v/v) fetal bovine serum (FBS; FCS-SA, Biosera) only. All incubation steps were performed at 23°C in sealed plastic bags. The threshold for detection of the antigen in ELISA was determined from control means (2 x TCM absorbance values). These values were consistently in the range of 0.050-0.100. Consequently, absorbance values ≥0.100 were considered as positive for the detection of antigen.

### Determination of Ig class and sub-cloning procedure

The Ig class of mAbs was determined by using antigen-mediated indirect ELISA (Thornton, 2001). Wells of microtiter plates coated with 20 μg EPS/mL PBS were incubated successively with hybridoma TCS for 1 h, followed by goat anti-mouse IgG1, IgG2a, IgG2b, IgG3, IgM or IgA-specific antiserum (ISO-2, Sigma) diluted 1:3000 in PBST for 30 min, and rabbit anti-goat peroxidase conjugate (A5420, Sigma) diluted 1:1000 for a further 30 min. Bound antibody was visualised with TMB substrate as described. Hybridoma cell lines were sub-cloned three times by limiting dilution, and cell lines were grown in bulk in a non-selective medium, preserved by slowly freezing in FBS/dimethyl sulfoxide (92:8 v/v), and stored in liquid N_2_.

### Antibody purification and enzyme conjugation

Hybridoma TCS of mAb TG11 was harvested by centrifugation at 2,147 x *g* for 40 min at 4°C, followed by filtration through a 0.8 μM cellulose acetate filter (10462240, GE Healthcare Life Sciences, UK). Culture supernatant was loaded onto a HiTrap Protein A column (17-0402-01, GE Healthcare Life Sciences) using a peristaltic pump P-1 (18-1110-91, GE Healthcare Life Sciences) with a low pulsation flow of 1 mL/min. Columns were equilibrated with 10 mL of PBS, and column-bound antibody was eluted with 5 mL of 0.1 M glycine-HCl buffer (pH 2.5) with a flow rate of 0.5 mL/min. The buffer of the purified antibody was exchanged to PBS using a disposable PD-10 desalting column (17-0851-01, GE Healthcare Life Sciences). Following purification, the antibody was sterile filtered with a 0.24 µm syringe filter (85037-574-44, Sartorius) and stored at 4°C. Antibody purity was confirmed by SDS-PAGE and gel staining using Coomassie Brilliant Blue R-250 dye (Thermo Fisher Scientific). Protein A-purified mAb TG11 was conjugated to horseradish peroxidase (HRP) for ELISA studies using a Lightning-Link horseradish peroxidase conjugation kit (701-0000; Bio-Techne Ltd.), or to alkaline phosphatase (AKP) for western blotting studies using a Lightning-Link alkaline phosphatase conjugation kit (702-0010; Bio-Techne Ltd.).

### Production of antigen *in vitro*

For antigen production studies, fungi were grown in liquid YNB+G medium for 72 h at 30°C with shaking (100 rpm) using the method described previously (Davies and Thornton, 2022). Culture fluids were filtered through Miracloth, and then stored at -20°C prior to immunoassay by western blotting and direct ELISA.

For colony blots, MEA was inoculated centrally with 5 μL of a 10^3^ spores/mL spore suspension of *L. corymbifera* (strain CBS109940) and incubated for 16 h at 30°C, after which the colony was overlayed with PVDF membrane (162-0175, Bio-Rad) for 8 h to bind extracellular antigens. The membrane was removed and discarded, the colony incubated for a further 16 h, and the blotting procedure repeated. The membrane was blocked and processed with TG11-AKP conjugate as described for western blotting.

### Polyacrylamide gel electrophoresis and western blotting

Sodium-dodecyl-sulphate-polyacrylamide gel electrophoresis (SDS-PAGE) was carried out using 4–20% gradient polyacrylamide gels (4561094, Bio-Rad) under denaturing conditions. Antigens in EPS preparations and culture filtrates were separated electrophoretically at 165 V, and pre-stained markers (1610377, Bio-Rad) were used for molecular weight determinations. For western blotting, separated antigens were transferred electrophoretically onto a PVDF membrane for 2 h at 75 V, and the membrane was blocked for 16 h at 4°C in PBS containing 1% (w/v) BSA. Blocked membranes were incubated with TG11-AKP conjugate diluted 1:15,000 (v/v) in PBS containing 0.5% (w/v) BSA (PBSA) for 2 h at 23°C. Membranes were washed three times with PBS, once with PBST and bound antibody visualised by incubation in substrate solution. Reactions were stopped by immersing membranes in dH_2_O, and membranes were then air dried between sheets of Whatman filter paper.

Monoclonal antibodies JF5 (Thornton, 2008), MC3 (Morad et al., 2018), ED7 (Al-Maqtoofi and Thornton, 2016) and HG12 (Thornton, 2009) were used as controls to confirm the presence of extracellular immuno-reactive antigens in *Aspergillus*, *Candida*, *Fusarium* and *Scedosporium* culture filtrates and EPS preparations, respectively. The anti-glucuronoxylomannan (GXM) mAb 18B7 (MABF2069, Sigma) was used to confirm the presence of immuno-reactive capsular polysaccharide of *Cryptococcus neoformans*.

### Direct ELISA

For direct ELISA, wells containing immobilised antigen were incubated with 50 µL of a 1:2000 (v/v) dilution of TG11-HRP conjugate in PBST for 1 h followed by TMB substrate solution for 30 min. All washing steps were as described for the indirect ELISA.

### TG11 lateral-flow device

#### LFD configuration

The TG11 Competitive lateral-flow device (TG11-LFD) was manufactured by Lateral Dx (Alloa, Scotland, UK). The test consisted of Kenosha 75 mm backing card; 8950, 222, and 1281 conjugate, top and sample pads, respectively; and a CN95 (12 μm) nitrocellulose membrane. The test (T) line consisted of EPS from the *L. corymbifera* strain, CBS109940, at a concentration of 0.5 mg/mL, while the internal test control (C) line consisted of goat anti-mouse IgG (Arista Biologicals) at a concentration of 0.25 mg/mL.

#### LFD specificity

Specificity of the TG11-LFD was determined using purified EPS preparations and with filtrates from 72-h-old YNB+G shake cultures of mucoralean and non-mucoralean yeasts and molds of clinical importance.

For EPS, 100 μL of running buffer (PBS containing 0.1% (v/v) Tween-20) containing 50 μg/mL of EPS was added to the TG11-LFD, and T and C line intensities were recorded after 30 min as artificial units (a.u.) using a Cube reader (Davies and Thornton, 2022). The threshold value for test positivity using EPS was determined from the negative control (running buffer only), which was consistently ≥400 a.u. Consequently, a T line value below the threshold value (400 a.u.) showed a positive test result.

For culture filtrates, samples were mixed 1:10 (v/v) with running buffer and 100 μL was added to the TG11-LFD. The intensities of the T and C lines were recorded after 30 min as artificial units (a.u.) as described. The threshold value for test positivity was determined from the T line values of culture filtrates for non-Mucorales yeasts and molds, which were consistently ≥400 a.u. Consequently, a T line value below the threshold value (400 a.u.) showed a positive TG11-LFD test result. The *Rhizopus arrhizus*-specific KC9-LFD (Davies and Thornton, 2022) was used for specificity comparisons, with the threshold value for test positivity determined from the T line values of culture filtrates from fungi other than *R. arrhizus*. These values were consistently ≥700 a.u. Consequently, a T line value below the threshold value (700 a.u.) showed a positive KC9-LFD test result.

#### LFD tests with human serum and BAL

Normal serum from healthy AB blood group males (H6914, Sigma) was spiked with purified EPS from the *L. corymbifera* strain, CBS109940, and was stored at -20°C prior to use. On thawing, the serum was mixed 1:1 (v/v) with citrate-dextrose solution (ACD; 22 g/L sodium citrate (C3434, Sigma), 7.3 g/L citric acid (C0759, Sigma) and 24.5 g/L D-(+)-glucose, pH~5.0), heated at 100°C for 5 min in a heating block, and then centrifuged for 5 min at 14,000 x *g*. The clear supernatant was mixed 1:1 (v/v) with serum running buffer (SRB; PBS containing 0.05% (v/v) Tween-20 and 0.05% (v/v) Triton X-305). One hundred-μL was added to the TG11-LFD, and the intensities of the T and C lines were recorded after 30 min as artificial units (a.u.) using the Cube reader.

Normal BAL from a healthy 59-year-old male (HUMANBAL-0101312, BioIVT) was spiked with purified EPS from the *Rhizopus arrhizus* var. *arrhizus* strain, CBS112.07, and was stored at -20°C prior to use. On thawing, the BAL was mixed 1:10 (v/v) with SRB and the resultant 100 μL containing 100 μg EPS/mL was added to the TG11-LFD. Normal BAL mixed 1:10 (v/v) with SRB, and SRB alone, acted as negative controls, with the intensities of the T and C lines recorded after 30 min as artificial units (a.u.) using the Cube reader.

### Statistical analysis

Numerical data were analysed using a Student’s t-test (independent, two-tailed) to determine statistical significance.

## Results

### Production of hybridomas and mAb isotyping

A single hybridoma fusion was performed, and 420 hybridoma cell lines were tested in indirect ELISA tests for recognition of the immunogen. Ten cell lines produced EPS-reactive antibodies of the immunoglobulin classes G2b (IgG2b) or M (IgM). The cell line TG11 (an IgG2b) was selected for further evaluation due to its isotype and broad recognition of Mucorales fungi, but lack of cross-reactivity with non-Mucorales fungi.

### Production of antigen *in vitro*

**Figure 1:**
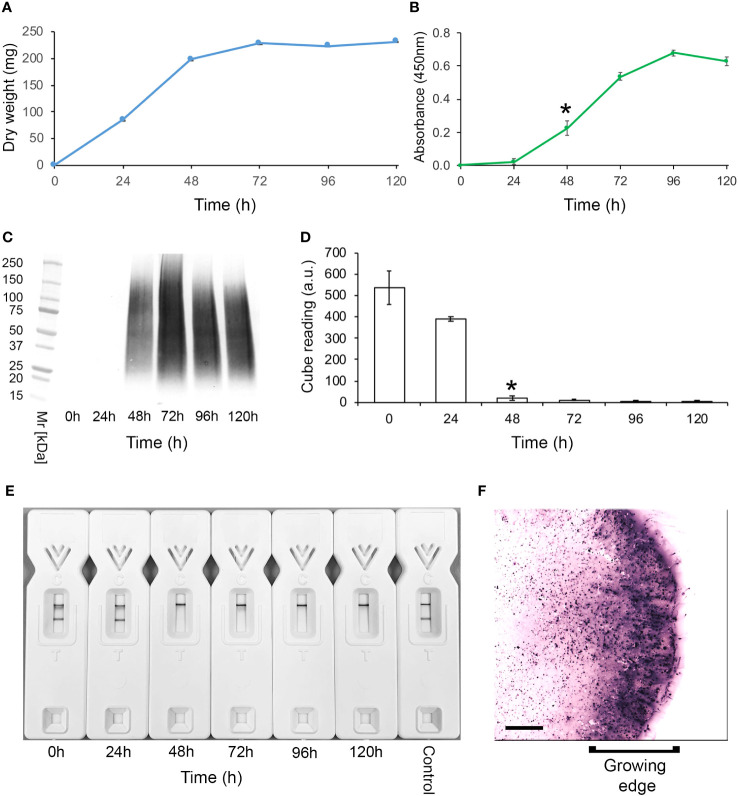
Production of the TG11 antigen by *Lichtheimia corymbifera* CBS109940. **(A)** Dry weights of the pathogen over a 5-day experimental period. **(B)** ELISA and **(C)** western blots of culture filtrates using mAb TG11, with a significant (Student’s t-test [*p* < 0.05]) increase in ELISA absorbance value at 48 h (indicated by *) compared to t = 0 h. **(D)** TG11-LFD test results using the culture filtrates, with test (T) line intensities measured as artificial units (a.u) using a Cube reader. There is a significant (Student’s t-test [*p* < 0.05]) reduction in T line intensity at 48 h (indicated by *) and thereafter, corresponding to the displacement of T lines in LFD tests at 48h, 72h, 96h and 120h **(E)**. The control **(E)** comprised running buffer (PBS containing 0.1% (v/v) Tween-20) only. Data points **(A, B)** and bars **(D)** are the means of 3 replicates ± SE. All LFD tests had control **(C)** line scores of >600 a.u. using the Cube reader. **(F)** Colony immunoblot showing extracellular production of the TG11 antigen. Note the intense immuno-staining of extracellular antigen produced at the growing edge of the colony during active growth of the pathogen. Scale bar = 1cm.

A study of antigen production by *Lichtheimia corymbifera*, strain CBS109940, in YNB+G shake culture showed that growth of the pathogen plateaued after 72 h (**Figure 1A**). Immunoassay of culture filtrates showed that the TG11 antigen was secreted into the culture medium and was first detectable by ELISA (**Figure 1B**), western blot (**Figure 1C**) and TG11-LFD (**Figures 1D, E**) 48 h post-inoculation. In colony immuno-blots, extracellular production of the TG11 antigen was associated with the growing edge of the colony (**Figure 1F**).

### Specificity of mAb TG11 in western blotting, LFD and ELISA immunoassay formats

#### Culture filtrates

**Figure 2:**
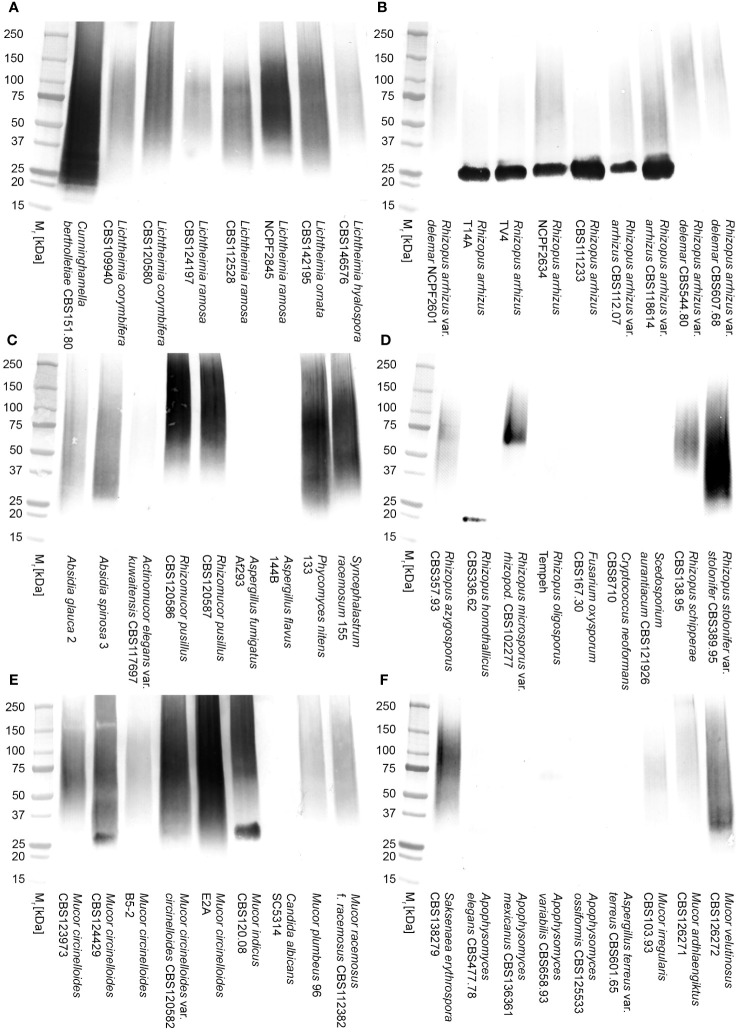
Western blots of culture filtrates from Mucorales fungi and from unrelated yeasts and molds of clinical importance using mAb TG11 **(A–F)**. The corresponding results for TG11-LFD and KC9-LFD tests of culture filtrates are shown in **Table 1**.

Western blotting studies of 72-h-old culture filtrates showed that mAb TG11 is specific to Mucorales fungi, binding to antigens with molecular weights of between 25 kDa to 250 kDa (**Figures 2A-F**), and with an additional immuno-reactive antigen of ~25 kDa in strains of *Rhizopus arrhizus* var. *arrhizus* (**Figure 1B**), *Mucor circinelloides* CBS124429 (**Figure 1E**) and *Mucor indicus* CBS120.08 (**Figure 1E**). A single immuno-reactive antigen of ~20kDa was evident in culture filtrates of *Rhizopus homothallicus* CBS336.62 (**Figure 2D**). There was weak binding with culture filtrates from *Rhizopus oligosporus* strain Tempeh (**Figure 2D**), *Apophysomyces elegans* CBS477.78 (**Figure 2F**) and *Apophysomyces variabilis* CBS658.93 (**Figure 2F**), and no binding to antigens in culture filtrates of *Apophysomyces ossiformis* CBS125533 or *Apophysomyces mexicanus* CBS136361 (**Figure 1F**). There was no cross-reaction of mAb TG11 with antigens in culture filtrates from the unrelated molds *Aspergillus fumigatus* strain Af293 and *Aspergillus flavus* strain 114B (**Figure 2C**), the yeast *Cryptococcus neoformans* CBS8710 (**Figure 2D**), the molds *Fusarium oxysporum* CBS167.30 and *Scedosporium aurantiacum* CBS121926 (**Figure 2D**), the yeast *Candida albicans* strain SC5314 (**Figure 2E**), and the mold *Aspergillus terreus* CBS601.65 (**Figure 2F**), despite the presence of extracellular immuno-reactive antigens in the culture filtrates of these fungi (**Supplementary Figure 1**).

In comparison to the KC9-LFD (Davies and Thornton, 2022), which was specific to antigens in culture filtrates of the *Rhizopus arrhizus* strains only (**Table 1**), the TG11-LFD is pan-Mucorales-specific, recognising antigens in culture filtrates from all of the Mucorales fungi tested (**Table 1**). There was displacement of the test (T) lines in all Mucorales TG11-LFD tests, with Cube reader values below the threshold value for test positivity (400 a.u.). There was no cross-reactivity of the TG11-LFD test with non-Mucorales yeasts and molds (**Table 1**), with a.u. values exceeding the 400 a.u. threshold value.

#### Extracellular polysaccharides

**Figure 3:**
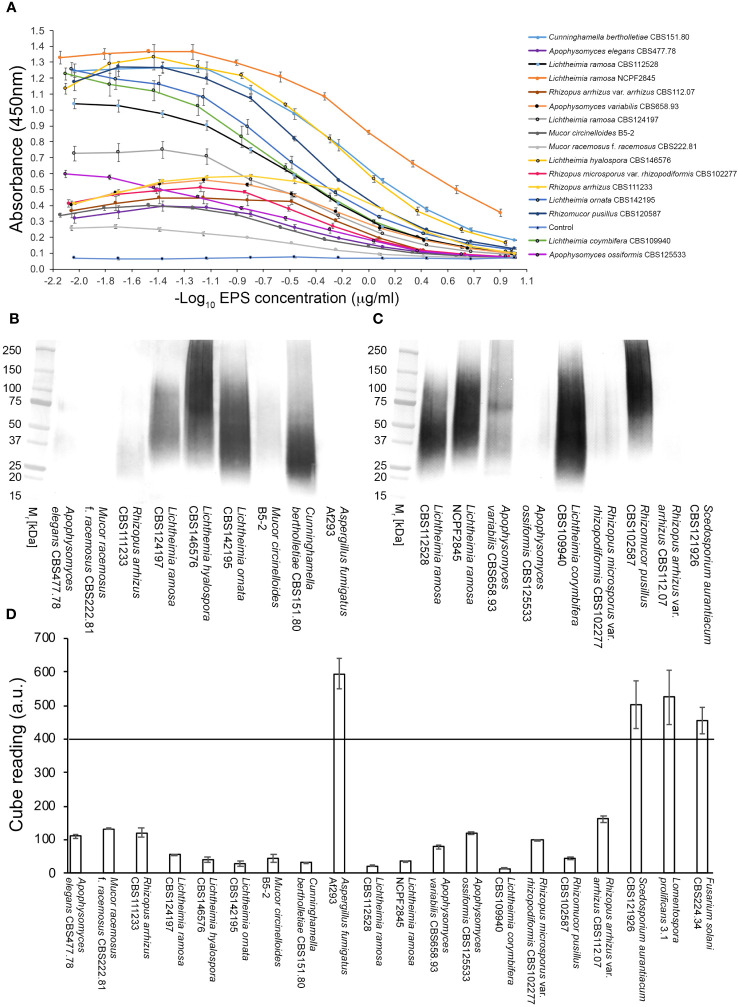
Reactivity of mAb TG11 with extracellular polysaccharide (EPS) from Mucorales spp. **(A)** ELISA of EPS samples from Mucorales species. The absorbance values of the negative control (phosphate buffered saline only) were consistently ≤0.100. Consequently, a threshold absorbance value of 0.100 was used for ELISA positivity. **(B, C)** Western blots of EPS samples (20 μg EPS/lane) from Mucorales spp. and from the unrelated human-pathogenic molds *Aspergillus fumigatus* Af293 and *Scedosporium aurantiacum* CBS121926. **(D)** Results of TG11-LFD tests using EPS samples (50 μg EPS/mL running buffer (PBS containing 0.1% (v/v) Tween-20)) from Mucorales spp. and from the unrelated molds *A. fumigatus* Af293, *S. aurantiacum* CBS121926, *Fusarium solani* CBS224.34, and *Lomentospora prolificans* strain 3.1. The threshold T line value for test positivity is ≤400 a.u. Values above this threshold show a negative LFD test result, while values below the threshold show a positive LFD test result. Data points **(A)** and bars **(D)** are the means of 2 replicates ± SE. All LFD tests **(D)** had control **(C)** line scores of >600 a.u. using the Cube reader.

In direct ELISA studies, mAb TG11 reacted with EPS preparations from all of the Mucorales fungi tested (**Figure 3A**), with absorbance values for all species exceeding the threshold value of 0.100 for test positivity. The sensitivity of the ELISA using EPS from the *L. corymbifera* strain CBS109940 was 109.8 pmol/L, with a range of 439.3 pmol/L to 54.9 pmol/L for the other Mucorales species tested. Western blotting studies with the EPS samples (**Figures 3B, C**) showed strong reactivity of mAb TG11 with EPS from *Lichtheimia* spp., *Cunninghamella bertholletiae* CBS151.80, *Rhizomucor pusillus* CBS102587, and *Apophysomyces variabilis* CBS658.93. Despite weaker reactions of mAb TG11 with the *Mucor* and *Rhizopus* EPS preparations in western blots (**Figures 3B, C**), EPS from all of the Mucorales fungi gave strong TG11-LFD test results (**Figure 3D**), with T line values below the threshold value (400 a.u.) for test positivity.

**Figure 4:**
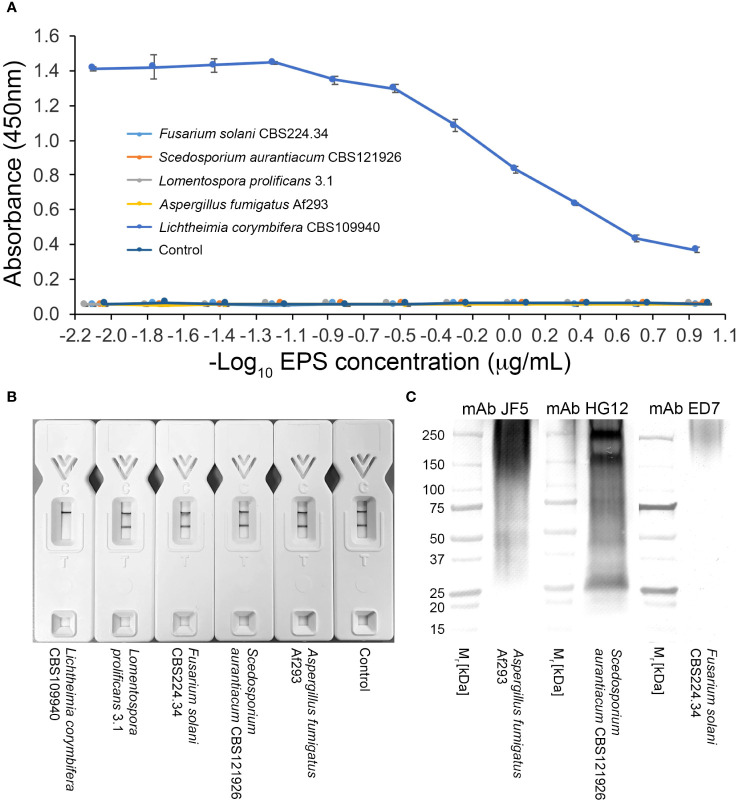
Specificity of mAb TG11. **(A)** ELISA of EPS samples from unrelated molds of clinical importance. Note the lack of cross-reactivity of mAb TG11 with EPS from the unrelated molds *A fumigatus* Af293, *S. aurantiacum* CBS121926, *Fusarium solani* CBS224.34 and *Lomentospora prolificans* strain 3.1, compared to the positive control (*Lichthemia corymbifera* CBS109940). Data points are the means of 2 replicates ± SE, and the negative control consisted of phosphate buffered saline only. **(B)** Visual appraisal of TG11-LFD specificity using EPS samples (50 μg EPS/mL running buffer (PBS containing 0.1% (v/v) Tween-20)) from *L. corymbifera* CBS109940 and the unrelated molds. Note the complete displacement of the test (T) line with *L. corymbifera* EPS compared to the unrelated molds and negative control (running buffer only). Using the Cube reader, all LFD tests had control **(C)** line scores of >600 a.u. **(C)** Western blots of EPS preparations (10 μg EPS/lane) from the human-pathogenic molds *A fumigatus* Af293, *S. aurantiacum* CBS121926 and *Fusarium solani* CBS224.34, showing the presence of antigens reactive with the *Aspergillus*-specific mAb JF5 (Thornton, 2008), *Scedosporium*-specific mAb HG12 (Thornton, 2009), and *Fusarium*-specific mAb ED7 (Al-Maqtoofi and Thornton, 2016), respectively.

There was no cross-reaction of mAb TG11 with EPS samples from the unrelated mold pathogens in either the western blotting (**Figures 3B, C**), LFD (**Figures 3D**, **4B**) or ELISA (**Figure 4A**) immunoassay formats, despite the presence of immuno-reactive antigens in these EPS preparations (**Figure 4C**).

#### Limit of detection (LOD) in human serum and compatibility with human BAL

**Figure 5:**
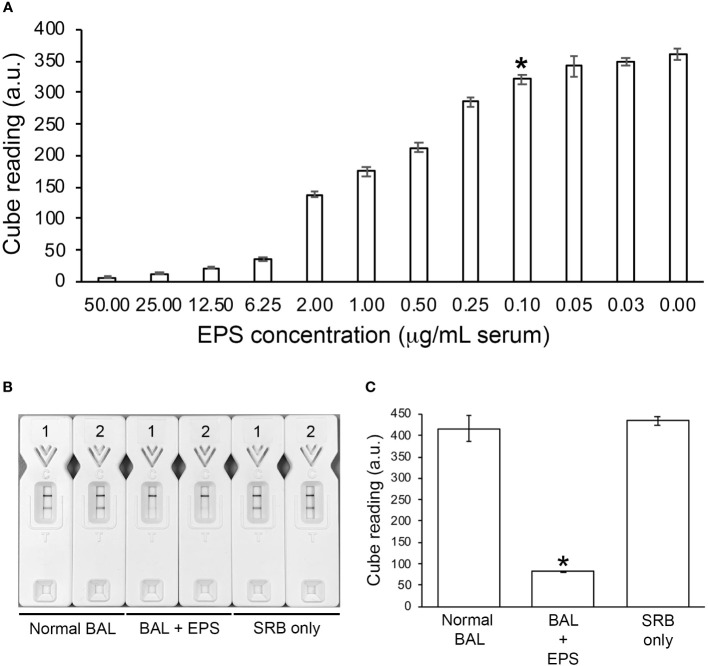
TG11-LFD tests with human serum and BAL. **(A)** Sensitivity of the TG11-LFD using *L. corymbifera* CBS109940 EPS diluted into human serum. Using the Cube reader, there were sequential decreases in test (T) line intensities with increase in EPS concentrations between 0.03 μg and 50.00 μg EPS/mL serum, with a significant reduction (Student’s t-test [*p* < 0.05]) at 0.10 μg EPS/mL serum (indicated by *) and thereafter compared to the control (normal serum only [0.00 μg EPS/mL serum]). Based on this result, the limit of detection (LOD) of the TG11-LFD is determined to be ~100 ng EPS/mL serum (~224.7 pmol/L serum). Data points are the means of 3 replicates ± SE, and all LFD tests had control **(C)** line scores of >600 a.u. **(B)** Visual appraisal of TG11-LFD compatibility with human BAL. Two replicate LFD tests with BAL samples spiked with 100 μg/mL EPS (BAL+EPS) from *Rhizopus arrhizus* var. *arrhizus* CBS112.07 are shown alongside replicate tests with normal BAL and serum running buffer (SRB) only. **(C)** Cube readings (a.u.) for the replicate LFD tests shown in **(B)**. Each value is the mean a.u. of the 2 replicate tests ± SE, and all LFD tests had control **(C)** line scores of >600 a.u. using the Cube reader. There is a significant (Student’s t-test [*p* < 0.05]) reduction (indicated by *) in the T line intensities of the BAL+EPS tests compared to both of the controls (normal BAL and SRB only), but no significant difference between the T line intensities of the two controls.

Using the Cube reader, the LOD of the TG11-LFD using human serum spiked with EPS from *L. corymbifera*, strain CBS109940, was shown to be ~100 ng EPS/mL serum (~224.7 pmol/L serum)(**Figure 5A**). The TG11-LFD is compatible with human BAL, showing a significant reduction in T line values using BAL spiked with EPS from the *Rhizopus arrhizus* var. *arrhizus*, strain CBS112.07, compared to the T line values for normal BAL and SRB only (**Figures 5B, C**).

## Discussion

In this paper, we describe the development and characterisation of a murine IgG2b monoclonal antibody (mAb), TG11, raised against an extracellular polysaccharide (EPS) antigen from *Lichtheimia corymbifera*, and its incorporation into a Mucorales-specific lateral-flow device (TG11-LFD) for detection of the EPS biomarker in human serum and BAL.

The antigen bound by mAb TG11 is secreted into culture medium following germination of sporangiospores, and is associated with active hyphal growth in colony immunoblots. Using a combination of immunoassay tests (ELISA, western blotting and LFD) of crude culture filtrates and purified EPS from Mucorales species and from unrelated molds and yeasts of clinical importance, we have shown that the mAb and the TG11-LFD are Mucorales-specific. Differences in reactivity of the mAb to antigens in crude culture filtrates and to purified EPS were observed in western blotting studies (for example, weak recognition of *A. variabilis* antigen in crude culture filtrate, but strong recognition of purified *A. variabilis* EPS). The reason for this discrepancy is currently unknown, but may be related to lower concentrations of antigen in the crude filtrates. Despite this, there was consistent recognition of antigens in both sources by the TG11-LFD, the intended clinical immunoassay format. All Mucorales fungi were detected, including the most common causes of mucoromycosis worldwide, namely *Lichtheimia* spp., *Mucor circinelloides*, *Rhizopus arrhizus*, and *Rhizopus microsporus* var. *rhizopodiformis* (Roden et al., 2005; Gomes et al., 2011; Skiada et al., 2011; Laternier et al., 2012; Zaki et al., 2014; Prakash and Chakrabarti, 2019; Skiada et al., 2020; Radotra and Challa, 2022; Özbek et al., 2023; Pham et al., 2023; Yang et al., 2023), and also rarer, emerging, or more regionally-prevalent causes of the disease (Gomes et al., 2011; Skiada et al., 2020) including *Actinomucor* (Tully et al., 2009), *Apophysomyces* (Chander et al., 2015), *Cunninghamella* (Mita et al., 2022), other *Mucor* spp. (Deja et al., 2006; Álvarez et al., 2011; Lu et al., 2013; Chander et al., 2018), *Rhizomucor* (Chander et al., 2018; Schober et al., 2021), other *Rhizopus* spp. (Anstead et al., 1999; Kokkayil et al., 2017; Chander et al., 2018; Kanwar et al., 2021), *Sakseneae* (Chander et al., 2017), and *Syncephalastrum* (Irshad et al., 2020). Importantly, mAb TG11 does not cross-react with other fungal pathogens which have been encountered in mixed infections with Mucorales spp., including *Aspergillus* spp. (Arndt et al., 2009; Bergantim et al., 2013; Henn et al., 2014; Obradovic-Tomasev et al., 2014; Pandey et al., 2019; Anita et al., 2021; Bellanger et al., 2021; Benhadid-Brahmi et al., 2022; Buil et al., 2021; Johnson et al., 2021; Moorthy et al., 2021; Paul et al. (2022); Ramírez-Hinojosa et al., 2021; Singh et al., 2021; Zayet et al., 2021; Rahna et al., 2022; Suresh et al., 2022; Tabarsi et al., 2022; Teng et al., 2022; Zhan et al., 2022; Murray et al., 2023; Sardeshmukh et al., 2023), *Fusarium* spp. (De Almeida Junior et al., 2015; Marino et al., 2023), *Candida albicans* (Obradovic-Tomasev et al., 2014; Pandey et al., 2019; Jawanda et al., 2022), *Cryptococcus neoformans* (Henn et al., 2014; Gupta et al., 2023), *Lomentospora prolificans* (Erami et al., 2023), and *Scedosporium apiospermum* (Shand et al., 2004; Marques et al., 2011; Song et al., 2022; Kanaujia et al., 2023).

The pan-Mucorales-specificity of mAb TG11 makes it a suitable candidate for incorporation into a rapid LFD test for detection of the Mucorales-specific EPS biomarker. While a pan-Mucorales mitochondrial rnl (encoding for large-subunit-ribosomal-RNA) gene has been shown to be a novel molecular marker for Mucorales fungi (Caramalho et al., 2019), and a Mucorales-specific IgM mAb (WSSA-RA-1) which binds to intracellular cytoplasmic antigens of between 14 kDa and 110 kDa has previously been reported and used in the immunohistochemical detection of bovine mucoromycosis (Jensen et al., 1996), this is the first time, to the best of our knowledge, that a pan-Mucorales-specific mAb which binds to an extracellular antigenic Mucorales biomarker has been reported. Previously, we described the development of a Competitive LFD for the serological detection of *Rhizopus arrhizus*, the principal global agent of mucoromycosis in humans. The species-specific LFD employs a mAb, KC9, which binds to a single epitope present within a 15 kDa extracellular EPS antigen (Davies and Thornton, 2022). We have found that mAb TG11 similarly binds to a single epitope within larger 25 kDa to 250 kDa Mucorales antigens, necessitating its use in a Competitive lateral-flow immunoassay format. The Competitive immunoassay format is ideally suited to mAbs which bind to single epitopes, with Competitive LFDs finding widespread applicability in human medicine, environmental sciences, agriculture, and veterinary medicine, for the detection of pathogens, hormones, enzymes, chemicals, narcotic drugs, toxins, and pollutants (Andryukov, 2020; Davies and Thornton, 2022; Khelifa et al., 2022).

The Competitive TG11-LFD test described here relies on soluble antigen in the test sample (buffer, serum or BAL) displacing binding of the gold-conjugated TG11 mAb to purified *L. corymbifera* EPS present in the test (T) line; the response is therefore negatively correlated to the analyte concentration (the more analyte present, the weaker the signal, with no analyte giving the strongest signal). An advantage of the Competitive LFD format is that it does not suffer from false-negative prozone effects caused by high concentrations of the target antigen as seen in Sandwich LFD formats. Indeed, the cryptococcal antigen (CrAg) semiquantitative (SQ) lateral-flow assay now comprises a Competitive test line to counteract prozone effects (Tadeo et al., 2021).

Current detection of mucoromycosis relies on insensitive and time-consuming culture of fungi from biopsy samples, and on sophisticated laboratory-based PCR, MALDI-TOF, or enzyme-linked immunospot (ELISpot) tests (Lackner et al., 2021; Lamoth, 2023; Thornton, 2023). Detection of mucoromycosis is not possible using the pan-fungal 1→3-β-D-glucan (BDG) test, since Mucorales fungi lack this carbohydrate in their cell walls. Nevertheless, the BDG test and *Aspergillus*-specific ELISA and LFD tests can be used to rule out aspergillosis, the most common differential diagnosis associated with mucoromycosis. The simplicity, speed, and low cost of LFDs makes them ideally suited to the detection of infectious diseases in low- to middle-income countries (Osaigbovo and Bongomin, 2021), and may aid in the point-of-care detection of mucoromycosis (Thornton, 2023). Currently, no mAb-based serodiagnostic LFD test exists for the specific detection of all infectious Mucorales fungi. A mAb (2DA6) and a lateral-flow immunoassay (LFIA) have been developed that recognise Mucorales species, but the mAb lacks specificity, cross-reacting with an epitope on α-1,6 mannans conserved among human pathogenic yeasts and filamentous fungi including *Candida albicans* and the angio-invasive moulds *Aspergillus*, *Fusarium*, and *Scedosporium* (Burnham-Marusich et al., 2018). Despite this, the LFIA was able to detect cell wall fucomannan in BALf, serum, and urine samples from diabetic ketoacidotic and neutropenic mice following intratracheal challenge with *Rhizopus delemar*, *Lichtheimia corymbifera*, *Mucor circinelloides* and *Cunninghamella bertholletiae* (Orne et al., 2018), demonstrating the utility of carbohydrate biomarkers in the diagnosis of mucoromycosis, and their detection using lateral-flow technology.

A hallmark of mucoromycosis is extensive angio-invasion (Spellberg et al., 2005; Ibrahim et al., 2012; Skiada et al., 2020), which presents an opportunity for serological detection of circulating EPS biomarker. We therefore investigated the compatibility of the TG11-LFD with human serum as a minimally-invasive biofluid for biomarker detection. When combined with a simple serum pre-treatment step, we were able to detect the EPS biomarker in spiked serum samples. Furthermore, using a Cube reader, we were able to determine the limit of detection (LOD) of *L. corymbifera* EPS as ~100 ng/mL serum (~224.7 pmol/L serum), which is within the range of sensitivities for Competitive lateral-flow immunoassays (Di Nardo et al., 2021). The relevance of this LOD has yet to be determined with samples from patients with ROCM, pulmonary, cutaneous or disseminated mucoromycosis, but compatibility of the TG11-LFD with human serum and BAL provides an opportunity for clinical evaluation of the test in different disease backgrounds (e.g. neutropenia, ketoacidosis, diabetes). When combined with the serum- and BAL-compatible *R. arrhizus*-specific LFD test (Davies and Thornton, 2022), the possibility exists for comprehensive point-of-care detection of all clinically-relevant Mucorales species.

## Data availability statement

The raw data supporting the conclusions of this article will be made available by the authors, without undue reservation.

## Ethics statement

Hybridoma work described in this study was conducted under a UK Home Office Project License, and was re-viewed by the institution’s Animal Welfare Ethical Review Board (AWERB) for approval. The work was carried out in accordance with The Animals (Scientific Procedures) Act 1986 Directive 2010/63/EU, and followed all the Codes of Practice which reinforce this law, including all elements of housing, care, and euthanasia of the animals. The study was conducted in accordance with the local legislation and institutional requirements.

## Author contributions

CRT: Conceptualization, Data curation, Formal Analysis, Funding acquisition, Investigation, Methodology, Project administration, Resources, Supervision, Validation, Visualization, Writing – original draft, Writing – review & editing. GED: Conceptualization, Data curation, Formal Analysis, Investigation, Methodology, Supervision, Visualization, Writing – review & editing. LD: Formal Analysis, Investigation, Writing – review & editing.

## Supplementary material

Supplementary Figure 1 Western blots of culture filtrates from non-Mucorales yeasts and molds of clinical importance showing the presence of **(A)** *Aspergillus* extracellular antigens reactive with the *Aspergillus*-specific mAb JF5 (Thornton, 2008), **(B)** *Scedosporium* extracellular antigens reactive with the *Scedosporium*-specific mAb HG12 (Thornton, 2009), **(C)** *Candida albicans* extracellular antigens reactive with the *Candida*-specific mAb MC3 (Morad et al., 2018), **(D)** *Fusarium* extracellular antigens reactive with the *Fusarium*-specific mAb ED7 (Al-Maqtoofi and Thornton, 2016), and **(E)** *Cryptococcus neoformans* capsular polysaccharide reactive with the anti-glucuronoxylomannan (GXM) mAb 18B7.
