# Supplementary figures and images for: Development of a monoclonal antibody and a lateral-flow device for the rapid detection of a Mucorales-specific biomarker
Source: Front Cell Infect Microbiol. 2023 Dec 8;13:1305662. doi: 10.3389/fcimb.2023.1305662 (PMC10739493; doi:10.3389/fcimb.2023.1305662)

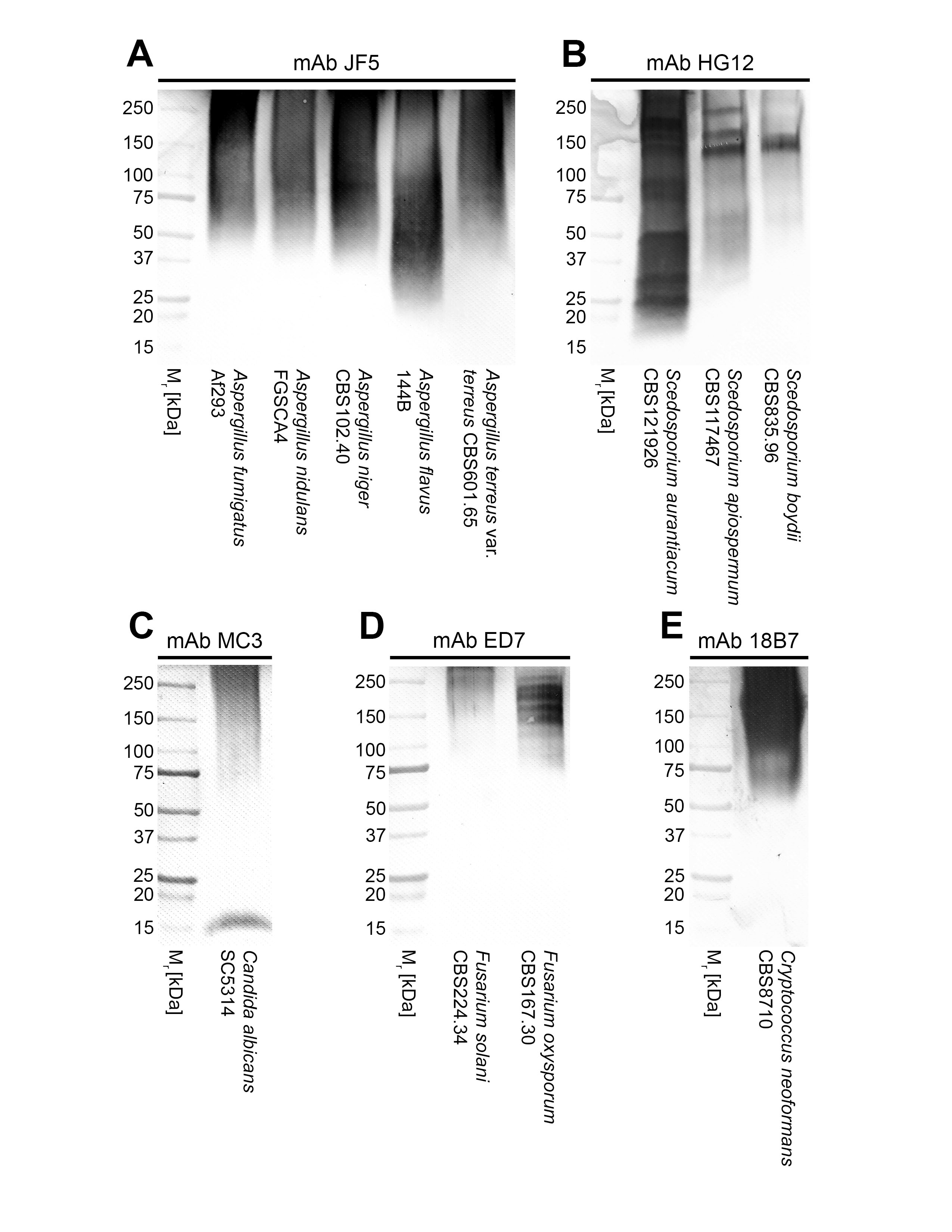

Supplement: Supplementary Figure 1 — Western blots of culture filtrates from non-Mucorales yeasts and molds of clinical importance showing the presence of (A) Aspergillus extracellular antigens reactive with the Aspergillus-specific mAb JF5 (Thornton, 2008), (B) Scedosporium extracellular antigens reactive with the Scedosporium-specific mAb HG12 (Thornton, 2009), (C) Candida albicans extracellular antigens reactive with the Candida-specific mAb MC3 (Morad et al., 2018), (D) Fusarium extracellular antigens reactive with the Fusarium-specific mAb ED7 (Al-Maqtoofi and Thornton, 2016), and (E) Cryptococcus neoformans capsular polysaccharide reactive with the anti-glucuronoxylomannan (GXM) mAb 18B7. [file Image_1.jpeg]
